# Lack of Usefulness of Donor-Derived Cell-Free DNA as a Biomarker for Cardiac Allograft Vasculopathy: A Prospective Study

**DOI:** 10.3389/fcvm.2022.856600

**Published:** 2022-04-06

**Authors:** Marta Jiménez-Blanco Bravo, Laura Pérez-Gómez, Francisco J. Hernández-Pérez, Carlos Arellano-Serrano, Mario Torres-Sanabria, Manuel Gómez-Bueno, Juan F. Oteo-Domínguez, Susana Mingo-Santos, Javier Segovia-Cubero

**Affiliations:** ^1^Hospital Universitario Puerta de Hierro Majadahonda, Madrid, Spain; ^2^Hospital Universitario Ramón y Cajal, Madrid, Spain; ^3^Centro de Investigación Biomédica en Red CIBER-CV, Instituto de Salud Carlos III, Madrid, Spain

**Keywords:** donor-derived cell free DNA, cardiac allograft vasculopathy, coronariography, biomarker, NTproBNP, cardiac troponin

## Abstract

**Background:**

Cardiac allograft vasculopathy (CAV) remains a major cause of morbidity and mortality among long-term heart transplant recipients. There is an unmet need for a non-invasive biomarker of CAV that could obviate the need to perform surveillance coronary angiograms in these patients. Our aim was to evaluate the performance of Donor-derived Cell Free DNA (dd-cfDNA) as a biomarker of CAV.

**Methods:**

We prospectively measured dd-cfDNA levels in all patients undergoing routine coronary angiography >1 year after heart transplant at a single center. Endpoints included the association between dd-cfDNA levels and the presence CAV, according to several prespecified criteria.

**Results:**

We included 94 heart transplant recipients, a median of 10.9 years after transplant. Coronary angiogram revealed CAV_0_, CAV_1_, CAV_2_, and CAV_3_ in 61, 19, 14, and 6% of patients, respectively. Comparison of dd-cfDNA levels in patients with CAV_0_ and CAV_1–2–3_ (primary end-point) did not show significant differences (0.92%, IQR 0.46–2.0 vs. 0.46%, IQR 0.075–1.5, *p* = 0.059), nor did the comparison between patients with stable CAV (no new coronary lesions since previous angiogram, *n* = 77) and progressive CAV (*n* = 17); dd-cfDNA values 0.735% (IQR 0.195–2.0) vs. 0.9% (IQR 0.12–1.8), *p* = 0.76. However, we found an association between NTproBNP levels and CAV degree (*p* = 0.017). Dd-cfDNA levels did not correlate with NTproBNP (ρ = −0.095).

**Conclusion:**

In this study, dd-cfDNA did not perform as a useful biomarker to avoid surveillance coronary angiograms for CAV diagnosis.

**Clinical Trial Notation:**

Potential Role of Donor-derived Cell Free DNA as a Biomarker in Cardiac Allograft Vasculopathy, NCT 04791852.

## Introduction

Cardiac allograft vasculopathy (CAV) remains the leading cause of long-term graft failure and a major cause of late death among heart transplant recipients (1). In spite of all the advances in the past years, its incidence has remained stable, affecting 25–30% of patients at 5 years and almost 50% after 10 years of transplant (2). In 2010, the International Society for Heart and Lung Transplantation (ISHLT) published a standardized consensus that classified it into four categories according to coronary angiography findings and cardiac allograft function (Supplementary Table 1) (3).

Owing to graft denervation, angina symptoms are very infrequent, and patients typically present with progressive heart failure or ventricular arrhythmias late in the course of the disease. Due to the poor prognosis it implies, it is of paramount importance to diagnose it at early stages, and clinical practice guidelines recommend an annual or biannual coronariography after heart transplant (4). However, coronary angiograms are an invasive technique with associated risks, and can cause significant patient discomfort. There is clearly a unmet need for a non-invasive biomarker of this entity that could obviate the need to perform surveillance coronary angiograms. Donor-derived Cell Free DNA (dd-cfDNA) has shown a good ability to rule out cellular rejection in heart transplant recipients (5–7), but its performance as a biomarker for CAV has not yet been tested.

The main objective of the FreeDNA-CAV study (Potential Role of Donor-derived Cell Free DNA as a Biomarker in Cardiac Allograft Vasculopathy, NCT 04791852) was to determine the ability of dd-cfDNA to detect asymptomatic CAV in a prospective cohort of heart transplant patients. Our main hypothesis was that allograft ischemia resulting from angiographic CAV would result in release of dd-cfDNA into the circulation.

## Materials and Methods

### Design

FreeDNA-CAV was a single center, observational, prospective, cross-sectional, investigator-driven study. We prospectively obtained dd-cfDNA levels in all consecutive asymptomatic patients who underwent surveillance coronary angiogram more than 1 year after an orthotopic heart transplant in our center between January 2019 and January 2021.

Main exclusion criteria were: age under 18 or over 80 years old, multiorgan transplant, estimated glomerular filtration rate <30 ml/min/m^2^, history of acute cellular rejection (ACR) ≥ 1R or antibody mediated rejection (AMR) in the previous 6 months, clinical suspicion of CAV (determined by the presence of heart failure, ventricular arrhythmias or ECG changes suggestive of myocardial ischemia), concomitant infection by Cytomegalovirus (CMV) or evidence of sepsis, inflammatory disease or neoplastic disease.

According to study protocol, all patients underwent on the same day: coronary angiogram, echocardiogram, electrocardiogram, blood sample extraction for dd-cfDNA quantification (%) and lab tests that included NT-proBNP, cardiac troponin, renal function, CMV PCR and anti-HLA antibodies, both donor-specific and non-donor specific (Luminex^®^ assay).

Echocardiogram was performed at the Imaging Unit of our center. Restrictive cardiac allograft physiology was defined as symptomatic heart failure with echocardiographic or hemodynamic suggestive findings according to ISHLT guidelines (3).

Concomitant acute rejection was ruled out by endomyocardial biopsy (EMB) in those patients who underwent surveillance angiography on the 12th month after heart transplant or after reduction of baseline immunosuppression (according to local protocol). On the rest of the cohort, acute rejection was assumed absent based on the lack of symptoms, normal echocardiogram and negative anti-HLA antibodies (Luminex^®^ assay). A cut-off value of median fluorescent intensity (MFI) < 3,000 was considered negative for this purpose.

Baseline immunosuppressive therapy in our center typically consists of a triple drug regimen, including Tacrolimus, Mycophenolate Mofetil (MMF) and low-dose prednisone. Steroid withdrawal is only performed in case of adverse events (approximately in 30% of patients) and is always monitored with periodic EMB. Statins are routinely prescribed in all heart transplant recipients, and anti-platelet agents are added in the presence of any degree of coronary disease.

The investigation conforms with the principles outlined in the Declaration of Helsinki. The study was approved by the local Institutional Review Board and all patients signed informed consent.

### End-Points

Our primary endpoint was the association between dd-cfDNA levels and the presence of any degree of CAV (CAV_0_ vs. CAV_1_, _2, or__3_), and to determine the discrimination ability of this biomarker in this situation using receiver-operator characteristics analysis.

Secondary end-points included association of dd-cfDNA with the different degrees of CAV (0, 1, 2, and 3), correlation of dd-cfDNA with NTproBNP and troponin I, and association of NTproBNP and troponin I with CAV presence and degree.

Two subgroup analysis were prespecified. First, patients were stratified by time since transplant into three groups: less than 5 years, 5–10 years and more than 10 years after heart transplant. Second, due to the insidious nature of CAV, a subgroup analysis according to the level of progression since the previous angiogram was performed. Patients with CAV_0_, and those with absence of new coronary lesions since previous angiogram were considered stable CAV, and patients with new coronary stenoses were considered progressive CAV for this purpose.

### Sample Processing and Quantification of %Donor-Derived Cell Free DNA

Test tubes for dd-cfDNA quantification were sent to Eurofins Megalab central laboratory in Madrid (Spain) *via* delivery courier on the same day of extraction. Once in there, two geneticists were in charge of registering the sample and checking that it met the requirements to be analyzed. Valid test tubes were then sent to Eurofins Genome laboratory in Rome (Italy), where the blood samples were processed and subsequently analyzed. All laboratory technicians and both genetists were blinded to the patient’s identity and the angiogram results.

The percentage of dd-cfDNA was measured using Next Generation Sequencing (NGS) technology, which measures differential allele contributions in a panel of amplified single nucleotide polymorphisms (SNPs) to quantify dd-cfDNA in recipients, avoiding the need to genotype the donor (8).

A panel of more than 500 SNPs with high heterozygosity, low amplification error, low linkage, is selected for amplification and sequencing. Cell-free (cfDNA) is extracted from 1 ml plasma and is then amplified using Ampliseq protocol (Thermo-Fisher). Amplicons are sequenced using S5 NGS sequencer (Thermo-Fisher). An analysis pipeline incorporating a custom Next Generation Sequencing bioinformatics tools is used to align reads to the SNPs regions and determine the contribution of donor-derived sequences and calculate the percentage of dd-cfDNA. The sequencing depth is >1,000 unique reads per sample, with an average of 4,000 reads. Eurofins Megalab reports the fraction of donor-derived cfDNA as a percentage, with values over 0.7% being considered positive based on previous studies on acute rejection (9).

### Coronary Angiograms

All coronary angiograms were performed at the cath lab in our institution by one experienced interventional cardiologist, who was blinded to the dd-cfDNA result. Coronary angiography results were classified according to ISHLT 2010 guidelines into four groups by the performing physician: CAV_0_, CAV_1_, CAV_2_, and CAV_3_ (3).

All studies were then reviewed by an independent interventional cardiologist, who acted as angiographic core lab and was blinded to the dd-cfDNA result and to the diagnosis made by the performing cardiologist. In case of disagreement with the original diagnosis, both interventional cardiologists reread the study, discussed the case and reached a consensus.

The angiographic core lab also reviewed previous angiograms and classified patients as stable CAV or progressive CAV, according to previously described criteria.

### Statistical Analysis

Statistical analysis was performed using Stata/IC software v16.1. (StataCorp (10), *Stata Statistical Software: Release 16*. College Station, TX: StataCorp LLC).

Categorical variables are presented as percentages and compared using the chi-square test or Fisher’s exact test. Numerical variables are presented as median and IQR, and compared using Kruskal-Wallis and U Mann–Whitney tests.

To test the discrimination ability of dd-cfDNA in CAV, an area under the curve-receiving operating characteristic (AUC ROC) was estimated. We considered a good AUC ROC when it was above 0.7. The correlation between dd-cfDNA and other biomarkers (NTproBNP and Troponin I) was tested by means of the Pearson correlation coefficient.

## Results

**FIGURE 1:**
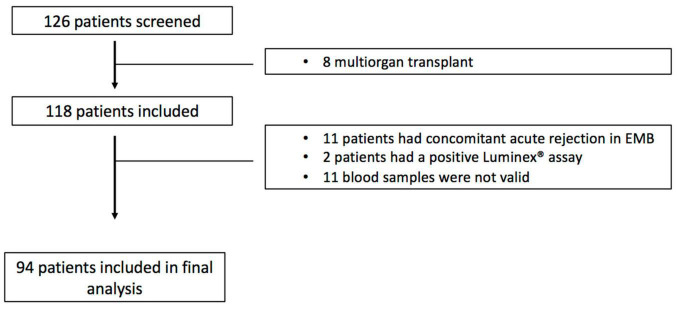
Study flow-chart.

From January 2019 to January 2021, a total of 126 heart transplant patients undergoing surveillance angiogram were screened for the study (Figure 1), and 94 patients were included in the final analysis.

Median age was 57 years (IQR 50–67), and 67% were men. Median time after heart transplant was 10.9 years (IQR 4.8–17.7). There were no statistically significant differences between patients with and without CAV regarding their baseline characteristics, except for NTproBNP levels and time after heart transplant, which were both significantly higher in patients with any degree of CAV. With respect to immunosuppressive treatment, patients with any degree of CAV were more likely to be on everolimus (14% vs. 40%, *p* = 0.005). Table 1 summarizes baseline characteristics of the cohort.

**TABLE 1:** Baseline characteristics of the cohort.

	Total (*n* = 94)	CAV_0_ (*n* = 57)	CAV_1_, CAV_2_ or CAV_3_ (*n* = 37)	*P*-value
Male sex, n (%)	63(67%)	37(64.9%)	26(70.3%)	0.589
Median age (IQR)	57(50−67)	56(47−70)	57(50−65)	0.728
Hypertension, n (%)	69(73.4%)	42(73.7%)	27(73%)	0.939
Diabetes mellitus, n (%)	32(34.0%)	23(40.4%)	9(24.3%)	0.109
Dyslipidemia, n (%)	48(51.1%)	30(52.6%)	18(46.2%)	0.706
BMI ≥ 30, n (%)	18(19.6%)	11(19.3%)	7(20.0%)	0.934
Median Creatinine levels, mg/dl (IQR)	1.11(0.95−1.42)	1.11(0.98−1.37)	1.13(0.91−1.52)	0.999
Median Estimated GFR, ml/min/1.72 m2 (IQR)	67(47−82)	66.5(50−80)	67(46−82)	0.631
Median NTproBNP, pg/ml (IQR)	401(228−934)	354(184−567)	673(318.5−1,616)	<0.01
Median Troponin I, μg/L (IQR)	0(0−0.02)	0(0−0.009)	0(0−0.02)	0.389
Echocardiogram				
Median LVEF,% (IQR)	60.5(56.8−65.2)	61(56.8−67.3)	60(56.3−62)	0.09
Median E/A (IQR)	1.85(1.6−2.25)	1.9(1.6−2.3)	1.8(1.5−2.1)	0.394
Median dd-cfDNA levels,% (IQR)	0.8(0.17−2)	0.92(0.46−2)	0.46(0.075−1.5)	0.059
Median time from heart transplant, years (IQR)	10.9(4.8−17.7)	9.8(4.1−13.6)	15.9(8.9−20.5)	0.004
Immunosuppressive treatment, n (%)				0.332
Calcineurin inhibitors	85(90.4)	54(94.8)	31(86.1)	0.148
MMF/Azatioprine	67(71.3)	44(77.2)	23(65.7)	0.230
Everolimus	22(23.4)	8(14.0)	14(40.0)	0.005
Prednisone	66(70.2)	42(77.8)	24(68.6)	0.005

*IQR, interquartile range; BMI, body mass index; GFR, glomerular filtration rate; LVEF, left ventricular ejection fraction; dd-cfDNA, donor-derived cell-free DNA; MMF, micophenolate mofetil.*

**FIGURE 2:**
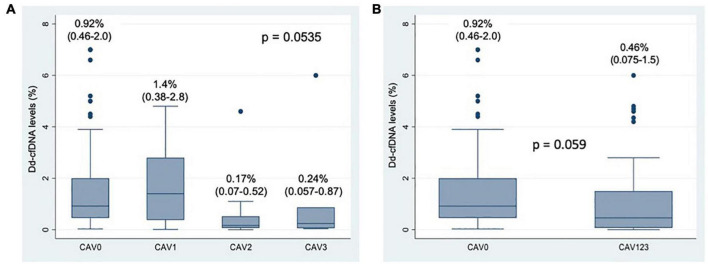
**(A)** Box-plot of Donor-derived Cell Free DNA levels according to CAV degree. Numerical data represent median dd-cfDNA values and their interquartile range. **(B)** Box-plot of Donor-derived Cell Free DNA levels according to CAV presence (CAV0 vs. CAV 1, 2, or 3). Numerical data represent median dd-cfDNA values and their interquartile range.

Coronary angiogram revealed CAV_0_ in 57 patients (61%), CAV_1_ in 17 patients (19%), CAV_2_ in 13 patients (14%) and CAV_3_ in 7 patients (6%); thus, there was a total of 37 patients (39%) with any degree of CAV. Median dd-cfDNA values for each CAV group are shown in Figure 2A.

The dd-cfDNA levels did not differ significantly between patients with and without CAV, *p* = 0.059 (Figure 2B).

**FIGURE 3:**
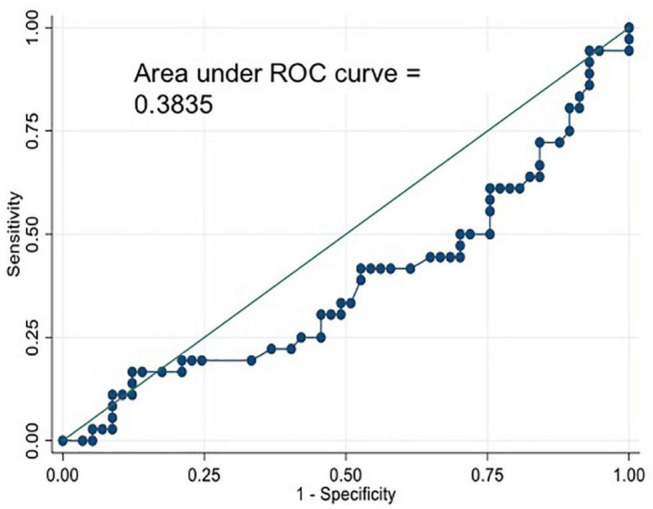
Area Under the Curve Receiver Operating Characteristics (AUC ROC) curve for the diagnosis of CAV0 vs. CAV123.

The AUC ROC curve for the diagnosis of CAV confirmed once more the lack of ability to predict the presence of any degree of CAV (AUC ROC = 0.38) (Figure 3).

### Subgroup Analysis

#### Time Since Transplant

**FIGURE 4:**
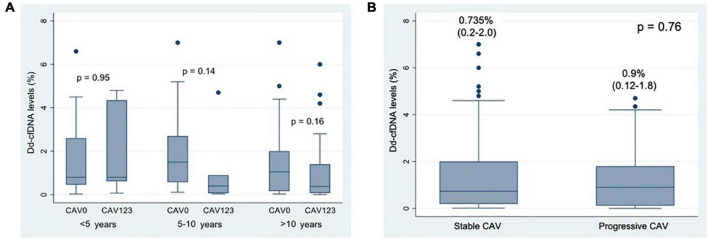
**(A)** Box-plot of Donor-derived Cell Free DNA levels according to time after heart transplant. **(B)** Box-plot of Donor-derived Cell-Free DNA according to CAV progression. Numerical data represent median dd-cfDNA values and their interquartile range.

There were no statistically significant differences between levels of dd-cfDNA in patients with or without CAV amongst the three prespecified subgroups: less than 5 years (*p* = 0.95), 5–10 years (*p* = 0.14) and more than 10 years after heart transplant (*p* = 0.16) (Figure 4A).

#### Cardiac Allograft Vasculopathy Progression

A total of 17 patients were classified as having progressive CAV (18%). No significant differences were found between patients with stable CAV (*n* = 77) and progressive CAV (*n* = 17), *p* = 0.76 (Figure 4B).

### Performance of Other Biomarkers and Correlation With Donor-Derived Cell Free DNA

**FIGURE 5:**
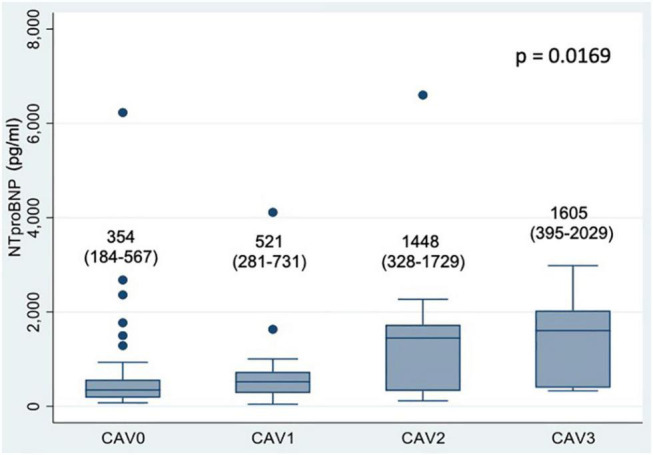
Box-plot of NTproBNP levels according to CAV degree. Numerical data represent median NTproBNP values (pg/ml) and their interquartile range. An outlier value of NTproBNP 26 350 pg/ml in the CAV_0_ group was excluded from the figure but included in the analysis.

We found a significant association between NTproBNP levels and increasing degrees of CAV (*p* = 0.0169). Median levels for each CAV group are shown in Figure 5. There was also a significant difference between CAV_0_ and CAV_1–2–3_ patients: 354 pg/ml (IQR 184–567) vs. 673 pg/ml (319–1,616), *p* < 0.01, with an AUC-ROC of 0.66 (Supplementary Figure 1). For an optimal cut-off point of 250 pg/ml, negative predictive value was 80%. There were no statistically significant differences between both groups in variables known to influence NT-proBNP levels, such as age, gender, obesity or renal insufficiency. No correlation was found between dd-cfDNA and NTproBNP levels (ρ = −0.095, *p* = 0.38).

A subset of 50 patients underwent Troponin I determination. We did not find an association between TnIc and the different degrees of CAV (*p* = 0.86), nor with dd-cfDNA levels (ρ = −0.096, *p* = 0.51).

## Discussion

To our knowledge, this is the first prospective study exploring the relation between dd-cfDNA and CAV. Even though we included a significant number of patients, and the incidence of CAV was similar to that described in previous registries (2), we could not find an association between the aforementioned biomarker and the presence or degree of CAV. Although, this could be interpreted as a “negative” study, we still think it is of great interest due to the unmet need of a non-invasive biomarker for this entity (11).

The presence of donor-specific DNA in plasma of solid organ transplant recipients was first described by Dennis Lo et al in 1998 (12). Since then, there has been an increasing interest in this technique, as the release of dd-cfDNA in the recipient’s blood secondary to cell damage in the graft makes these molecules potential biomarkers of graft health (13). Most of the research so far has been focused on its ability to rule out acute cellular rejection. However, a recent review by K Kush points out the need to explore its potential use as a biomarker for CAV (14).

The data published to date in this field is rather scarce. Holzhauser et al found a borderline significantly higher proportion of patients with CAV (defined as Stanford III-IV or angiographic disease) in the subset of patients with dd-cfDNA above the median (*p* = 0.047) (15). Of note, DSA were present in 27% of the high dd-cfDNA group. This means that AMR, a condition known to increase dd-cfDNA levels, could not be ruled out, which could potentially explain the difference with our results. On the other hand, another study performed in 66 pediatric patients using a method that does not require genotyping the donor, found that ddcf-DNA levels were not significantly higher in samples associated with CAV (0.27% vs. 0.55%, *p* = 0.057) (16). These findings are in line with our results. The ongoing SHORE registry (NCT 03695601) will hopefully add more data to the question of a correlation between dd-cfDNA and CAV.

The evidence for dd-cfDNA as a biomarker of acute cellular rejection (ACR) in heart transplant recipients has already been established (5, 6).

Therefore, it seemed reasonable to speculate that allograft ischemia resulting from CAV would result in release of dd-cfDNA into the circulation. However, the pathophysiology of CAV differs from that of ACR. Some studies have implied immune-mediated pathways (chronic immune response, acute rejection) whereas others have involved non-inmunological factors, such as classical cardiovascular risk factors or CMV infection (17, 18).

This multifactorial etiology could potentially explain the results of our study. Even though CAV is associated with graft damage, this injury is more insidious and episodic than that of acute rejection. Moreover, CAV is clinically silent until late stages, averting the recognition of critical time-points in which necrosis occurs. Our hypothesis is that dd-cfDNA rises only during subclinical acute ischemic episodes, in the same way as cardiac troponin. Both are not usually elevated in patients with chronic ischemic cardiomyopathy. Unfortunately, the design of our study does not allow to draw inferences about the performance of the biomarker throughout the different stages of the disease.

Overall dd-cfDNA values in our study were higher than those reported in previous studies using slightly different techniques (5, 6). However, our median time after heart transplant was 10.9 years, and there is no evidence yet on the “normal” values of this biomarker at that stage post-transplant. In any case, due to the fact that all samples were tested in the same laboratory and with the same method, our conclusions should still be valid.

We chose to evaluate the performance of dd-cfDNA only in asymptomatic patients because it is in this subset of patients where the biomarker would be of most utility. We feel that, in symptomatic patients or with high suspicion of CAV, it would be advisable to perform an angiogram regardless of the levels of dd-cfDNA. However, this design choice (made on clinical grounds) might have reduced the effect size and, there, the power of our study to detect a significant association between dd-cfDNA and coronary allograft vasculopathy. Thus, this might partly explain the “negative” results of this study.

Neither of the subgroup analyses (by CAV severity and time since transplantation) revealed a meaningful relation with dd-cfDNA.

### Correlation With Other Biomarkers

Our study showed a significant association between NTproBNP and the presence and severity of CAV. In spite of its wide availability, very few studies have focused on natriuretic peptides as biomarkers of CAV. In the study by Mehra et al., BNP was associated with the development of CAV (defined as coronary artery stenosis ≥ 40%), and cardiac deaths were significantly more prevalent in the subset of patients with BNP ≥ 250 pg/ml (19). The study by Arora et al confirmed the prognostic value of NTproBNP in heart transplant recipients, but only predicted CAV when combined with C-reactive protein (20).

Even though in our series we found a good correlation between NTproBNP levels and the degree of CAV, its ability to rule out the disease was suboptimal, with a AUC-ROC below 0.7. Sensitivity and specificity were only fair, and we could not find a negative predictive value cut-off of use in clinical practice.

On the other hand, we measured cardiac Troponin I in a subset of patients, but no relationship between this biomarker and CAV could be found. This supports our hypothesis that patients with angiographic CAV do not seem to have active myocardial necrosis during long periods of time.

Evidence regarding cardiac Troponin I as CAV biomarker is rather scarce. Labarrere et al found that patients with persistently elevated levels of this biomarker during the 1st year post-transplant had a significant higher risk for subsequent development of CAV (OR 4.3, *p* < 0.001) (21). However, there is no solid evidence relating Troponin I and coronary angiogram findings during late follow-up.

### Limitations

This study has several limitations that must be taken into account. First of all, it is a cross-sectional study, so the kinetics of the biomarker during the development of CAV could not be studied. However, our main goal was to test the ability of dd-cfDNA as a substitute for surveillance coronary angiograms in asymptomatic patients, and in this sense our study appropriately addresses this question.

Secondly, we did not perform routine intravascular ultrasound (IVUS) in our study, limiting our ability to relate the biomarker with earlier stages of the disease. Correlation with IVUS would have required studies during the first post-heart transplant year, in which intimal changes with prognostic significance occur. Nonetheless, coronary angiograms is the preferred method for CAV surveillance due to its wider availability and proven prognostic value.

Last, but not least, only a minority of patients underwent simultaneous endomyocardial biopsy. Nevertheless, the presence of acute cellular rejection in the absence of clinical suspicion in a cohort of asymptomatic patients a median of more than 10 years after heart transplant is exceptional, and the performance of routine endomyocardial biopsy in this setting is not usual in our clinical practice.

## Conclusion

In this single center study, donor-derived cell-free DNA was not associated with the presence of CAV. The search for a biomarker with a high negative predictive value that could obviate the need to perform periodic surveillance angiograms is still open.

## Data Availability Statement

The raw data supporting the conclusions of this article will be made available by the authors, without undue reservation.

## Ethics Statement

The studies involving human participants were reviewed and approved by the Comité Etico de Investigación con Medicamentos del Hospital Universitario Puerta de Hierro Majadahonda. The patients/participants provided their written informed consent to participate in this study.

## Author Contributions

MJ-B and JS-C participated in the research design, the writing of the manuscript, and data analysis. LP-G participated in the performance of the research and data analysis. FH-P and MG-B participated in the research design and the performance of the research. CA-S, MT-S, JO-D, and SM-S participated in the performance of the research. All authors contributed to the article and approved the submitted version.

## Conflict of Interest

The authors declare that the research was conducted in the absence of any commercial or financial relationships that could be construed as a potential conflict of interest.

## Publisher’s Note

All claims expressed in this article are solely those of the authors and do not necessarily represent those of their affiliated organizations, or those of the publisher, the editors and the reviewers. Any product that may be evaluated in this article, or claim that may be made by its manufacturer, is not guaranteed or endorsed by the publisher.
