# Supplementary material for: Lack of Usefulness of Donor-Derived Cell-Free DNA as a Biomarker for Cardiac Allograft Vasculopathy: A Prospective Study
Source: Front Cardiovasc Med. 2022 Apr 6;9:856600. doi: 10.3389/fcvm.2022.856600 (PMC9019134; doi:10.3389/fcvm.2022.856600)
Supplement: Supplementary file 1 [file Data_Sheet_1.pdf]

SUPPLEMENTARY MATERIAL

LACK OF USEFULNESS OF DONOR-DERIVED CELL-FREE DNA AS A BIOMARKER FOR CARDIAC ALLOGRAFT VASCULOPATHY (FREEDNA-CAV): A PROSPECTIVE STUDY

Figure S1. Area Under the Curve Receiver Operating Characteristics (AUC ROC) curve for the diagnosis of CAV0 vs CAV123 using NT-proBNP.

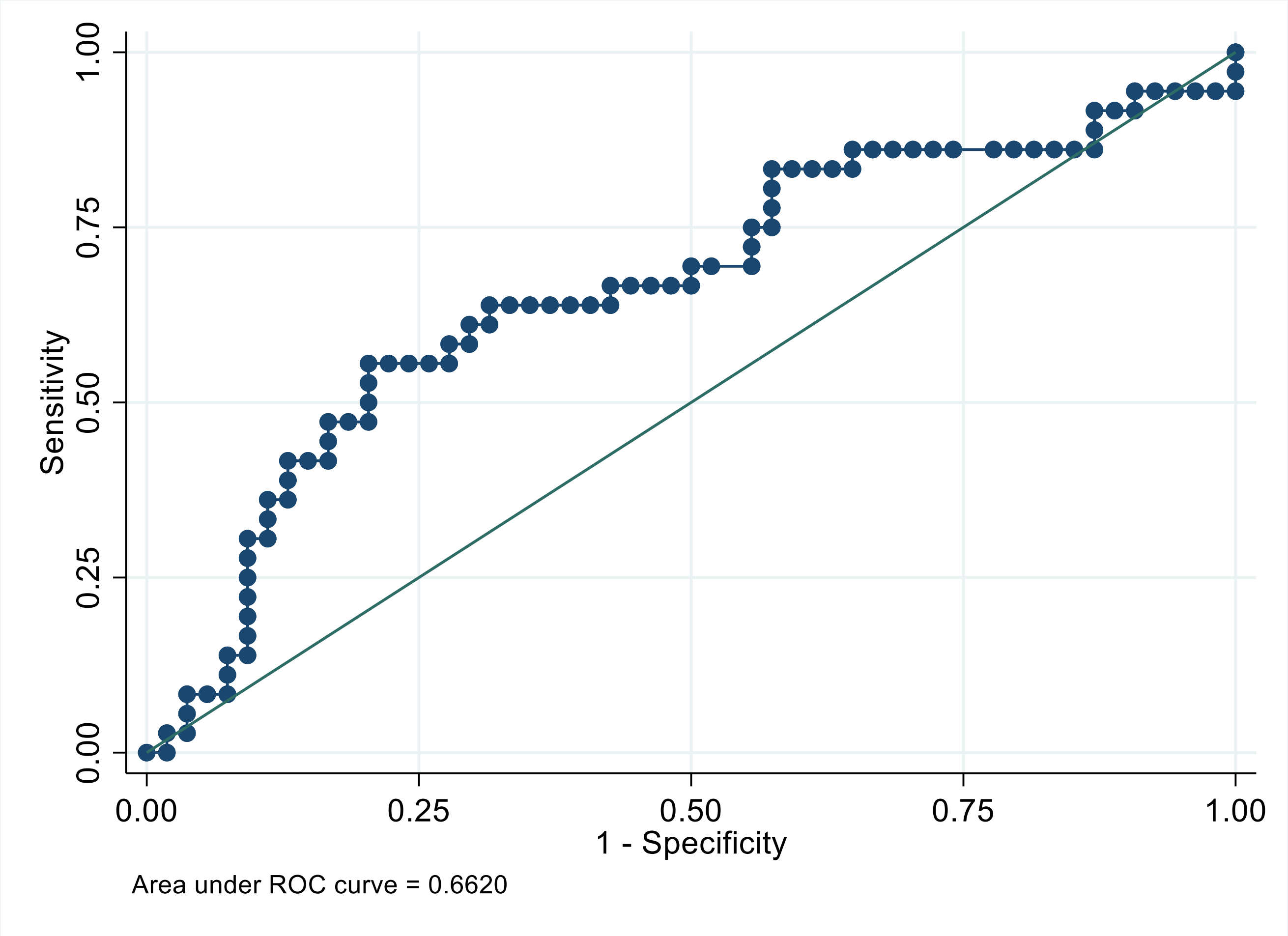

Table S1. Recommended Nomenclature for Cardiac Allograft Vasculopathy (adopted from Mehra et al, 2010[3])

ISHLT CAV_0 (not significant)	No detectable angiographic lesion
ISHLT CAV_1 (mild)	Angiographic left main (LM) < 50%, or primary vessel with maximum lesion of < 70%, or any branch stenosis < 70% (including diffuse narrowing) without allograft dysfunction
ISHLT CAV_2 (moderate)	Angiographic LM < 50%; a single primary vessel >= 70%, or isolated branch stenosis >= 70% in branches of 2 systems, without allograft dysfunction
ISHLT CAV_3 (severe)	Angiographic LM >= 50%, or two or more primary vessels >= 70% stenosis, or isolated branch stenosis >= 70% in all three systems; or ISHLT CAV_1 or CAV_2 with allograft dysfunction (defined as LVEF <= 45% usually in the presence of regional wall motion abnormalities) or evidence of significant restrictive physiology (defined as symptomatic heart failure with echocardiographic E to A velocity ratio > 2, isovolumetric relaxation time < 60 msec, deceleration time < 150 msec or restrictive hemodynamic values, right atrial pressure > 12 mmHg, Pulmonary Capillary Wedge Pressure > 25 mmg and Cardiac Index < 2 l/min/m2)

A “Primary Vessel” denotes the proximal and middle 33% of the left anterior descending artery, the left circumflex, the ramus and the dominant of co-dominant right coronary artery with the posterior descending and posterolateral branches.

A “Secondary Branch Vessel” includes the distal 22% of the primary vessels or any segment within a large septal perforator, diagonals and obtuse marginal branches or any portion of a non-dominant right coronary artery.
